# Platelet-Rich Plasma and Adipose-Derived Mesenchymal Stem Cells for Regenerative Medicine-Associated Treatments in Bottlenose Dolphins (*Tursiops truncatus*)

**DOI:** 10.1371/journal.pone.0108439

**Published:** 2014-09-24

**Authors:** Richard J. Griffeth, Daniel García-Párraga, Maravillas Mellado-López, Jose Luis Crespo-Picazo, Mario Soriano-Navarro, Alicia Martinez-Romero, Victoria Moreno-Manzano

**Affiliations:** 1 Centro de Investigación Príncipe Felipe, Tissue and Neuronal Regeneration Lab, Valencia, Spain; 2 Oceanogràfic (grupo Parques Reunidos), Valencia, Spain; 3 Centro de Investigación Príncipe Felipe, Electron Microscopy Unit, Valencia, Spain; 4 Centro de Investigación Príncipe Felipe, Cytomics Unit, Valencia, Spain; 5 FactorStem, Ltd. Valencia, Spain; National Institutes of Health, United States of America

## Abstract

Dolphins exhibit an extraordinary capacity to heal deep soft tissue injuries. Nevertheless, accelerated wound healing in wild or captive dolphins would minimize infection and other side effects associated with open wounds in marine animals. Here, we propose the use of a biological-based therapy for wound healing in dolphins by the application of platelet-rich plasma (PRP). Blood samples were collected from 9 different dolphins and a specific and simple protocol which concentrates platelets greater than two times that of whole blood was developed. As opposed to a commonly employed human protocol for PRP preparation, a single centrifugation for 3 minutes at 900 rpm resulted in the best condition for the concentration of dolphin platelets. By FACS analysis, dolphin platelets showed reactivity to platelet cell-surface marker CD41. Analysis by electron microscopy revealed that dolphin platelets were larger in size than human platelets. These findings may explain the need to reduce the duration and speed of centrifugation of whole blood from dolphins to obtain a 2-fold increase and maintain proper morphology of the platelets. For the first time, levels of several growth factors from activated dolphin platelets were quantified. Compared to humans, concentrations of PDGF-BB were not different, while TGFβ and VEGF-A were significantly lower in dolphins. Additionally, adipose tissue was obtained from cadaveric dolphins found along the Spanish Mediterranean coast, and adipose-derived mesenchymal stem cells (ASCs) were successfully isolated, amplified, and characterized. When dolphin ASCs were treated with 2.5 or 5% dolphin PRP they exhibited significant increased proliferation and improved phagocytotic activity, indicating that in culture, PRP may improve the regenerative capacity of ASCs. Taken together, we show an effective and well-defined protocol for efficient PRP isolation. This protocol alone or in combination with ASCs, may constitute the basis of a biological treatment for wound-healing and tissue regeneration in dolphins.

## Introduction

Dolphins exhibit an extraordinary capacity to heal deep soft-tissue injuries, such as those following shark bites [1]. Dolphins in captivity often experience external soft tissue injuries as a result of repetitive exercises and movements, such as open wounds on the underside of their lower mandible which slowly develop and worsen during training regimens. Often times, dolphins which were injured in the open ocean are rescued and rehabilitated in captivity [1]. Nevertheless, accelerated wound healing in wild or captive dolphins may help minimize infection and other side effects associated with open wounds in marine animals.

Platelet-rich plasma (PRP) is a fraction of plasma with a higher number of platelets compared to whole blood, thereby containing increased concentrations of growth factors [2]. The alpha granules in platelets are the source of multiple growth factors including vascular endothelial growth factor (VEGF), platelet-derived growth factor (PDGF), and transforming growth factor beta (TGFβ) among others [3]. These growth factors play an essential role in the complex processes of wound healing and tissue regeneration [4]. PRP stimulates type 1 collagen, matrix metalloproteinase 1, and increases the expression of regulators of cell cycle progression to accelerate wound healing [5], [6], and has been widely used in many species, including humans, for regenerative medicine in an increasing variety of surgical fields. Successful clinical applications have been reported using PRP for wound repair, soft tissue healing [2], [7], cosmetric surgery [8]–[10], burns [11], nervous tissue [12], [13], chronic skin ulcers [14], maxillofacial and long bone defects as well as in the treatment of joints in various mammals [3], [15]–[18]. However, some studies have suggested that PRP had little or no benefit, which most likely was the result of poor quality PRP [16], [19], [20]. Nevertheless, PRP has already been used in a wide variety of applications for regenerative medicine purposes. Moreover, at this point there is no agreed upon gold standard protocol for PRP generation and little characterization has been performed on the obtained products. Often times protocols vary across and within species, including the use of protocols defined for certain species being used for others without any additional characterization [21]. Well-defined simple procedures will result in very useful therapeutic tools, especially for veterinary medicine. The optimization of centrifugation conditions is fundamental to obtaining high quality PRP with minimal manipulation. Quantification and identification of platelets and lymphocytes as well provides a proper characterization of the PRP concentration procedure. Additionally, maintaining platelet integrity and quality without damaging or lysing them allows them to fully secrete growth factors upon controlled activation. Furthermore, PRP treatment enhances angiogenesis [22] and stimulates stem cell proliferation and cell differentiation for tissue regeneration [9]. Undifferentiated stem cells migrate to the site of growth factors delivered from PRP applications and trigger proliferation of the stem cells at the site [15].

Mesenchymal stem cells are an attractive cell population for regeneration of musculoskeletal tissues and wound healing [23]–[25]. Multiple sources of mesenchymal stem cells have been described including bone marrow, ligaments, lung, umbilical cord, and adipose tissue [26]. Adipose-derived stem cells (ASC) in particular are an appealing source because of their abundant availability and excellent ability to expand and proliferate in culture. In humans, ASC have been used successfully to treat soft tissue defects, scars, and burn injuries and to regenerate various damaged tissues [10]. Recently, ASCs from dolphins have been isolated, cultured, and differentiated into adipogenic, chondrogenic and osteogenic cell lineages [27], thereby demonstrating that dolphin ASCs may have similar regenerative potential as other already documented mammals. Here we have defined a simple and well-characterized protocol for efficient isolation of both ASCs and PRP in dolphins. The use of PRP separately or in combination with ASCs has the potential to provide a safe and efficient treatment for soft tissue injuries and regeneration not previously described in this species.

## Materials and Methods

### Animals and blood collection

Blood samples were collected from the tail vein plexus from 9 different dolphins at a local aquarium (Oceanografic; http://www.cac.es/oceanografic) for routine hematological and biochemical testing. To prevent clotting, whole blood was collected into tubes containing sodium citrate and the excess blood (∼10 ml) from each dolphin was used for these studies. In accordance with the European Parliament and Council normative 2010/63/UE (22nd September 2010) on the protection of animals used for scientific purposes and with the Real Decreto 53/2013 (1st February 2013), under the standards for the protection of animals used for experimental and other scientific purposes including teaching, “The non-experimental clinical veterinary practice” (RD 53/2013 Article 2, section 5) is excluded from the scope of the legislation and therefore approval from the corresponding ethical committee was not required. The Centro de Investigación Príncipe Felipe (CIPF) and the Oceanografic have a signed collaborative agreement for research purposes. Under this agreement CIPF obtained consent, specifically for the use of surplus dolphin blood collected for routine general exams. As part of the preventative medical care program, blood samples are taken every two months from these dolphins. The excess blood collected was used for this study. Whole blood from adult male and female bottlenose dolphins (*Tursiops truncatus*) between the ages of 7–25 years were utilized. Blood was transported to the adjacent laboratory at 4°C immediately and processed within 30 min after collection. To compare samples of dolphin blood with those of human blood, surplus blood samples from healthy anonymous donors from a local blood donation program (Unidad de Transfusiones de la Comunidad Valenciana; http://centro-transfusion.san.gva.es) were collected and processed separately but identically to that of dolphin blood. Human blood samples had been donated for use in transfusions, however after a certain time in storage, these samples were no longer recommended for transfusion and this blood was to be discarded and destroyed. Therefore, we were able to take advantage of this surplus blood for use in research and accordingly informed consent for the use of this blood for research purposes was not required.

### Centrifugation of blood samples and PRP isolation

**Figure 1:**
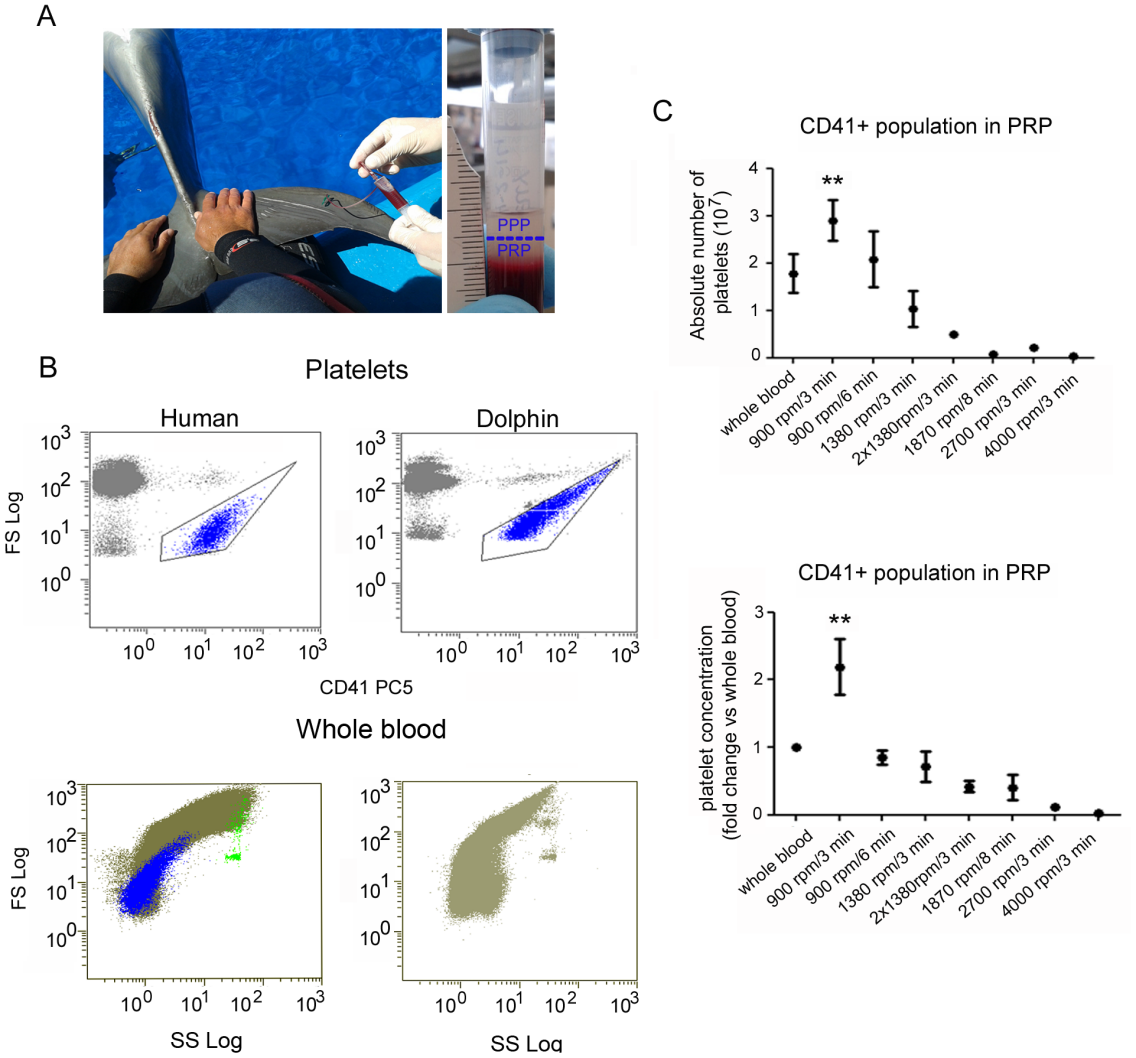
Efficient dolphin platelet-rich plasma concentration protocol. (A) Adult male and female bottlenose dolphins (*Tursiops truncatus*) between the ages of 7–25 years were utilized. Blood samples were collected from the tail vein plexus from 9 different dolphins at a local aquarium and placed into tubes containing sodium citrate. After centrifugation the upper half of the plasma was considered platelet-poor plasma (PPP) and discarded while the lower half was considered platelet-rich plasma (PRP) and used for subsequent experiments. (B) Representative images of FACS analysis utilizing a human CD41 antibody which recognized human and dolphin platelets (upper panels); Acquisition profile of FS versus SC of both, human and dolphin whole blood are shown in lower panels (in blue is represented the CD41 positive population in human sample). (C) Whole blood samples were subjected to multiple centrifugation protocols to determine which was the most efficient in concentrating platelets in a small volume of plasma. Significant increases in absolute number of platelets and platelet concentration as determined by fold change compared to whole blood were observed when whole blood samples were centrifuged at 900 rpm for 3 min. Asterisks denote a significant difference compared to whole blood; ** *P*<0.01.

Equal volume (1 ml) of whole blood samples in tubes containing sodium citrate were gently inverted multiple times before centrifugation. Following centrifugation, the plasma fraction was divided into two parts. The upper half was considered platelet-poor plasma and removed while the lower half was considered platelet-rich plasma and used for further analysis (Figure 1A). Centrifugation was performed at room temperature in an Eppendorf 5810R centrifuge with a swing-bucket rotor (A-4–62, Eppendorf) at the following centrifugation speeds (and equivalent forces) and durations: 1) one spin at 900 rpm (equivalent to 106× g) for 3 min, 2) one spin at 900 rpm (equivalent to 106× g) for 6 min, 3) one spin at 1380 rpm (250× g) for 3 min, 4) two spins at 1380 rpm (250× g) for 3 min each (this consisted of a first spin at 1380 rpm (250× g). The bottom half of the plasma fraction was collected and then spun again at 1380 rpm (250× g) for an additional 3 min and the bottom half of this fraction was considered PRP and used for analysis), 5) one spin at 1870 rpm (460× g) for 8 min, 6) one spin at 2700 rpm (958× g) for 3 min, and 7) one spin at 4000 rpm (2102× g) for 3 min.

### FACS analysis

To determine the concentration of platelets in whole blood and in PRP fractions, the human cell platelet marker CD41 was utilized (BD Bioscience, USA). For absolute numbers of platelets, BD Trucount Tubes (BD Bioscience, USA) were used and the fold change of platelet concentration in each PRP fraction was compared to whole blood. Fifty µl of whole blood or 50 µl of each PRP fraction were incubated with 10 µl CD41 antibody conjugated with PC5 (BD Bioscience, USA) for 30 minutes at room temperature in the dark. Subsequently, 20 µl of this mix was then transferred to the BD Trucount Tubes and diluted with 1 ml of PBS and used for FACS analysis. Flow cytometry acquisition and analysis was performed on a FC500 flow cytometer (Beckman Cultek, USA). For absolute platelet number the following formula was utilized:
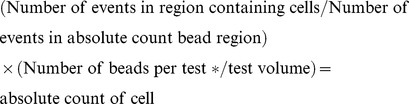
*Number of beads per test: 52250

For ASC characterization, cell suspension after passage 2 was assayed for cell surface protein expression of CD90-PE, CD44-PE-Cy7, CD105-PE, CD34-PE-Cy5 and CD45-FITC (BD Pharmigen, USA). Cells were trypsinized and pelleted, resuspended in PBS at a concentration of 10^5^ cells/100 µl, and incubated at a 1∶100 dilution for each antibody or alone for background controls. Cells were incubated in the dark for 45 min at room temperature and then washed three times with PBS and resuspended in 0.5 ml of PBS for FACS analysis. The mean ± SD of the 2 different tested samples were determined for each condition, ASCs cultured in the presence of autologous serum (10% dolphin serum) or cultured with 10% fetal bovine serum (FBS).

### Transmission electron microscopy

PRP fractions were fixed in 2.5% glutaraldehyde in 0.1M phosphate buffer (PB) for 1 hr. Then, the cells were washed with 0.1M PB three times and a single drop of 1.5% agar was added. Sections were post-fixed with 2% osmium, rinsed, dehydrated and embedded in Durcupan resin (Fluka, Sigma-Aldrich, St. Louis, USA). Semithin sections (1.5 µm) were cut with an Ultracut UC-6 (Leica, Heidelberg, Germany) and stained lightly with 1% toluidine blue. Finally, ultra-thin sections (0.08 µm) were cut with a diamond knife, stained with lead citrate (Reynolds solution) and examined under a transmission electron microscope FEI Tecnai G2 Spirit (FEI Europe, Eindhoven, Netherlands) attached to a digital camera Morada (Olympus Soft Image Solutions GmbH, Münster, Germany). To quantify the area, diameter, and absolute number of alpha granules Image J software was utilized. The mean ± SD of 3 different tested samples were determined for dolphin and human PRP.

### Quantification of growth factors

Activation of platelets is required to release the growth factors and was performed by adding 14.3units/ml thrombin and 1.4 mg/ml CaCl_2_ to the PRP samples (human n = 5, dolphin n = 7), followed by incubation at 37°C for 1 hr. Non-activated PRP was included as a negative control, in this case no reactivity was detectable for any tested growth factor. The samples were then centrifuged at 4000× g for 10 min at room temperature and the supernatant was collected and stored at −80°C until growth factor quantification by enzyme-linked immunosorbent assay (ELISA), which was performed using Luminex xMAP Technology. Affymetrix kits (eBioscience) containing antibodies against human PDGF-BB, VEGF-A, and TGFβ were utilized according to the manufacturer's instructions and detection of growth factors was performed on a Luminex 200 system and analyzed using Exponent 3.1 software by extrapolating the absolute value from the standard curve for each growth factor.

### Adipose tissue extraction, ASC isolation and cell culture

The Oceanografic aquarium is part of the Stranding Network through an agreement between the “Ciudad de las Artes y las Ciencias” and the “Conselleria de Infraestructuras, Territorio y Medio Ambiente”. Through this agreement both institutions have transferred to the Oceanografic the rights for veterinary assistance in cases of stranded sea turtles and cetaceans. This agreement includes the rights to euthanize animals when required and perform autopsies in collaboration with the University of Valencia. The agreement also allows for the use of samples from the cadaveric tissue for research purposes. Once the health and condition of the animal(s) are evaluated, if euthanasia is required, the guidelines for euthanasia of non-domestic animals are followed [28]. Additionally, in accordance with the European Parliament and Council normative 2010/63/UE (22nd September 2010) and the Real Decreto 53/2013 (1st February 2013) in post-mortem tissue collection for research purposes, approval from the corresponding ethical committee is not required. In this study adipose tissue was not collected from live dolphins, therefore approval from the corresponding ethical committee was not required for the development of research-related studies from post-mortem animals.

On two separate occasions in 2013 the veterinary team at the Oceanografic in Valencia, Spain were notified about a stranded wild striped dolphin (*Stenella coeruleoalba*) found along the eastern Spanish Mediterranean coast. The first dolphin was already dead when encountered but the second dolphin was euthanized by intravenous administration of a lethal dose of pentobarbital [28], [29] by the veterinarians. This dolphin was not euthanized specifically for use in this study. Collection of adipose tissue from both dolphins was completely opportunistic. Stranded dolphins are occasionally found along the eastern Spanish Mediterranean coast and are assessed by veterinarians from the Oceanografic. In the case of the euthanized dolphin, the official clinical evaluation by the veterinarians indicated that rehabilitation was not possible. This dolphin was unable to swim or keep normal flotation and exhibited severe neurological-related abnormalities including tremors, convulsive episodes and loss of reflexes. Therefore, this dolphin was euthanized. Adipose tissue was opportunistically collected after confirmation of death by the official veterinarian and the local authorities.

In both instances, the recently postmortem (0±0.5 days) cadavers were transported to the Oceanografic and adipose tissue was obtained from the postnuchal fat pad, placed into a solution containing PBS plus antibiotic and transported to the adjacent laboratory. The adipose tissue was washed multiple times in PBS plus antibiotics to clean the tissue and remove residual blood. In a petri dish, 10 g of adipose tissue were added to a solution containing PBS, 100 units/ml penicillin and 100 µg/ml streptomycin (Gibco 15140) and collagenase type IA (0.07%, Sigma C9891 CA, USA) and the tissue was manually cut into small pieces using sterile surgical scissors in a laminar flow hood and digested overnight at 37°C, 20% O_2_, 5% CO_2_. The following day the digested adipose tissue was collected and washed multiple times with PBS plus antibiotic by centrifugation. The pellet was then resuspended in growth medium (DMEM medium containing 10% dolphin serum or 10% heat-inactivated FBS, 2 mM L-glutamine, 30% L-glucose, 100 units/ml penicillin and 100 µg/ml streptomycin), plated in petri dishes, and incubated overnight. The following day the medium was removed and replaced with fresh medium and attached cells were allowed to grow until nearly confluent then subsequently passaged three times and subjected to viability/proliferation assays, FACS analysis and phagocytosis assays.

#### ASC directed-differentiation

Once the ASC expanded in vitro and equivalently to previous procedures [27], passages after 4, were distributed to induce adipogenesis, osteogenesis and chondrogenesis differentiation process. All directed-differentiation mediums were obtained from Lonza catolog. *Adipogenesis*: ASC were seeded at a cell density of 10000 cells/cm^2^ and when ASC have became>90% confluence the growth medium is substitute for differentiation medium containing, among others, insulin, Dexamethasone, IBMX (3-isobutyl-methyl-xantine) and indomethacin (Adipose Derived stem cell Basal Medium; Lonza Group Ltd). The cells were then incubated for 10–12 days. The adipogenic differentiation was evaluated by Oil Red staining of the lipid vacuoles in formalin fixed cultures; *Osteogenesis*: ASC were seeded at a cell density of 10000 cells/cm^2^ in collagen I (Sigma; 10 mM) coated plates in medium containing among others 0.1 µM dexamethasone, 50 µM Asc2P and 10 mM μ-glycerophosphate (Osteogenic Basal Medium; Lonza Group Ltd) with 10% of fetal bovine serum (FBS). ASC cultures were maintained in this medium for 4 weeks (with medium changes every 3 days). For detection of extracellular calcium deposits the Alizarin Red staining was used in formalin fixed cultures; *Chondrogenesis*: The ASC culture was performed from cell “Micromass” starting form with a high concentration of cells in a minimal volume (1×10^5^ cels/100 µl) in the presence of TGF-β 1 and 3 10 ng/ml, Asc 2P (50 µM) and insulin (6.25 µg/ml) (Chondro BulletKit; Lonza Group Ltd) for four weeks with medium changes every 3 days. Alcian blue was used to detect the presence of enrichment of sulfated proteoglycans in the extracellular matrix. Before staining, the micromass cultures were fixed in formalin, included in paraffin and sectioned into 10 µm. All samples were carried out in parallel with or without additional 2.5% PRP in the corresponding differentiation mediums.

### ASC viability and proliferation

Briefly, 10^4^ dolphin ASCs at passage 3–4 were seeded in 96-well plates and allowed to grow for 24 hr in growth medium containing 10% FBS. Serum deprived growth medium was then supplemented with 50 U/ml heparin and dolphin ASCs were treated with 0, 1, 2.5 or 5% dolphin PRP or the same concentration of FBS as a positive control. All groups were then subjected to the cell viability test, CellTiter 96 AQueous Non-Radioactive Cell Proliferation Assay (MTS assay; Promega, CA, USA). Every condition was assayed in quadruplicate in three different experiments for both lines of dolphin ASCs. The viability of cells at each assayed condition was expressed as the percentage ratio of the mean ± SD of colorimetric signal from treated cells in the presence of 1, 2.5, or 5% PRP compared to the absence of PRP.

For phagocytosis assays, 10^5^ dolphin ASC at passage 3–4 were seeded into 35 mm petri dishes and allowed to grow for 24 hr in growth medium containing 10% FBS. Serum deprived growth medium was then supplemented with 50 U/ml heparin and dolphin ASCs were treated with 0 or 5% dolphin PRP in the presence of 2 µm diameter red fluorescent microspheres (Invitrogen F8826). After 24 hr incubation, the cells were fixed with 4% PFA, washed with PBS, and images were taken immediately. For Giemsa staining and morphological assessment, ASCs treated with microspheres were incubated for 24 h, then fixed in cold 100% methanol for 20 min, and then stained with Giemsa (Fluka, UK) for 1 hr. Giemsa was then removed and the ASCs were washed with tap water, allowed to air-dry, and images were taken immediately.

### Statistics

Statistical comparisons were assessed by Student's *t*-test. All *P* values were derived from a two-tailed statistical test using the Graphpad Prism 5 Software. A *P*-value<0.05 was considered statistically significant.

## Results

### Low centrifugation speed and short duration yield the highest quality PRP in bottlenose dolphins

Low centrifugation speed and short duration yielded the highest quality PRP preparation from dolphin whole blood (Figure 1). Following blood collection and centrifugation, the top half of the separated plasma was considered platelet-poor plasma (PPP) and removed, while the bottom half was considered PRP and utilized for the experiments in this study (Figure 1A). A human antibody against the platelet cell surface marker CD41 showed reactivity for dolphin platelets by FACS analysis, and a similar cell profile for human and dolphin whole blood was found by CD41 immunoreactivity (Figure 1B). An additional population of non-gated cells also showed positive labeling for CD41 but at a higher size (FS) corresponding to a small population of activated platelets that bind to a fraction of leukocytes. For absolute numbers of platelets, BD Trucount Tubes (BD Bioscience, USA) were used and the fold change of platelet concentration in each PRP fraction was compared to whole blood. Quantification of the dolphin CD41+ cell population revealed that the centrifugation protocol with the most enriched PRP was 900 rpm (equivalent to 106× g) for 3 min (Figure 1C, upper panel). There was a significant increase in the absolute number of platelets and a significant, more than a 2-fold increase, in the concentration of platelets in this fraction of PRP (Figure 1C, lower panel). Using the same centrifugal force but increasing the duration to 6 min caused a slight but insignificant decrease in the platelet concentration compared to whole blood while the absolute number of platelets was slightly but insignificantly increased compared to whole blood (Figure 1C). Increasing the centrifugation speed to 1380 rpm (250× g) with duration of 3 min resulted in a slight but insignificant decrease in platelet concentration and absolute number of platelets (Figure 1C). However, two sequential centrifugations at 1380 rpm for 3 min resulted in a significant decline in both absolute number of platelets and fold change compared to whole blood (Figure 1C). Three other conditions were evaluated, each with increasing centrifugal forces (1870 rpm = 460× g, 2700 rpm = 958× g, and 4000 rpm = 2102× g) and each resulted in significant decreases in absolute number of platelets and fold change compared to whole blood (Figure 1C).

### Dolphin platelets are larger than human platelets

**Figure 2:**
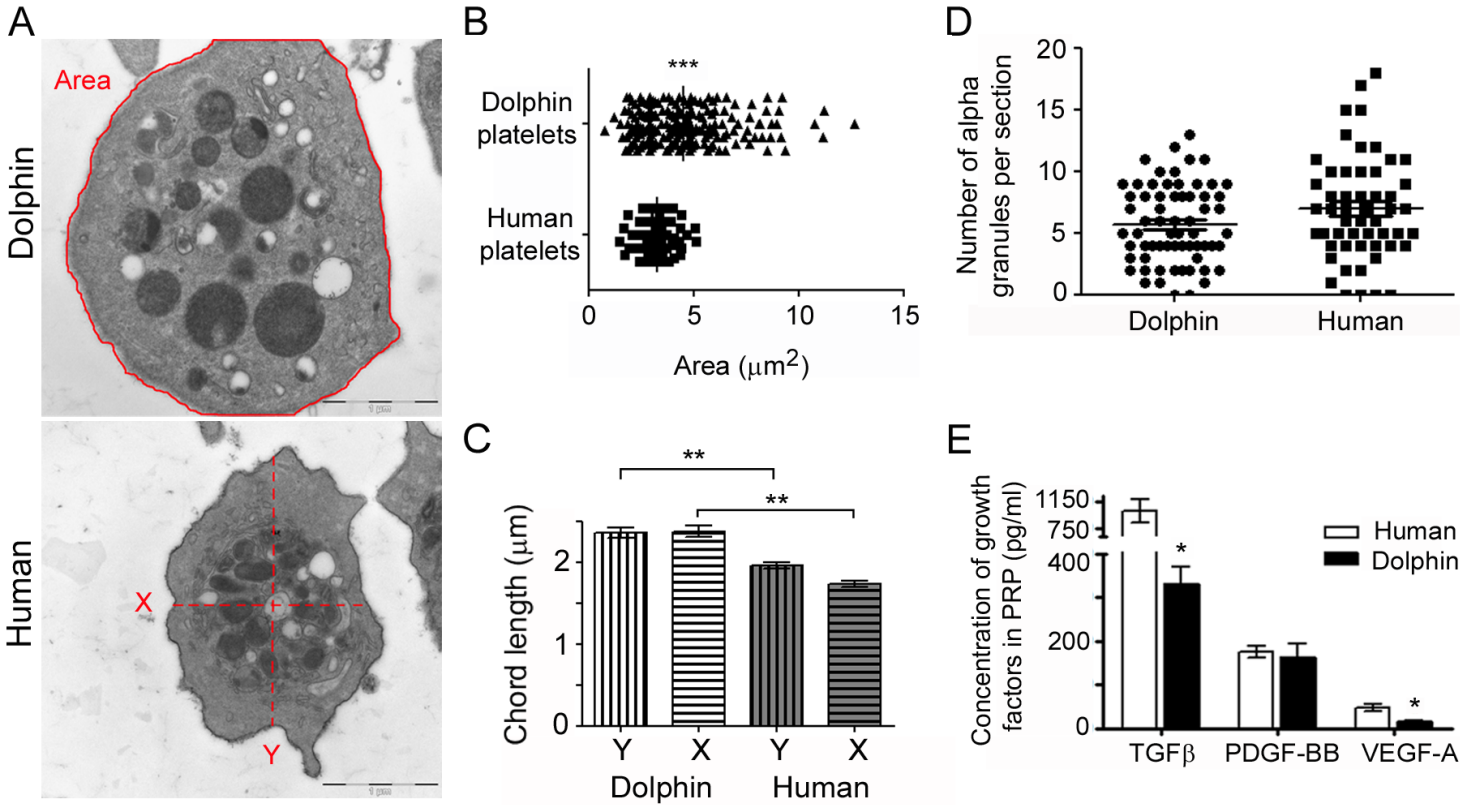
Characterization of dolphin platelets. (A) Representative images of the morphology of dolphin and human platelets by transmission electron microscopy revealed the larger size of dolphin platelets compared to human platelets. (B) Utilizing Image J software, the circumference of platelets that were sectioned through the major axis was traced and the area was calculated (see A upper panel). The area of dolphin platelets was significantly larger than those of human platelets. (C) Chords were drawn through the center of the platelet sections (approximate diameters) in the X and Y axes and these lengths were measured (see A lower panel). Chords from both axes were significantly longer in dolphin platelets than human platelets. (D) There were no significant differences in the number of alpha granules per section between dolphin and human platelets. Results presented in A–D are mean ± SEM of 3 human samples and 3 dolphin samples. A minimum of 50 measurements were taken from each sample. (E) The concentrations of TGFβ and VEGF-A were significantly reduced in dolphin PRP compared to human PRP, while there was no difference in the concentration of PDGF-BB. Results are mean ± SEM of 5 human samples and 7 dolphin samples. Asterisks denote significant differences; * *P*<0.05, ** *P*<0.01, *** *P*<0.001.

Transmission electron microscopy studies demonstrated that dolphin platelets have a significantly larger area compared to human platelets (Figure 2A–C). The mean area ± SEM of dolphin platelets was 4.53 µm^2^±1.8 while that of human platelets was 3.26 µm^2^±0.8. Likewise, linear measurements taken from sections cut through the major axis of nearly rounded elliptical platelets, were longer in dolphin platelets compared to human platelets (Figure 2A,C). However, similar numbers of alpha granules were found in cross sections of human platelets compared to dolphin platelets (Figure 2A,D).

### Quantification of TGFβ, PDGF, and VEGF in dolphin PRP

The concentrations of TGFβ and VEGF-A in dolphin PRP were significantly lower than that in human samples, while there were no differences in the concentration of PDGF-BB (Figure 2E). Mean concentrations ± SEM for five humans and seven dolphins were: TGFβ; human 1016±177 pg/ml, dolphin 331±37 pg/ml, PDGF-BB; human 174±13 pg/ml, dolphin 163±30 pg/ml, and VEGF-A; human 46±8 pg/ml, dolphin 14±2 pg/ml.

### Isolated Dolphin ASCs are plastic adherent, express mesenchymal-specific surface antigens and have the capacity for tri-lineage mesenchymal differentiation

**Figure 3:**
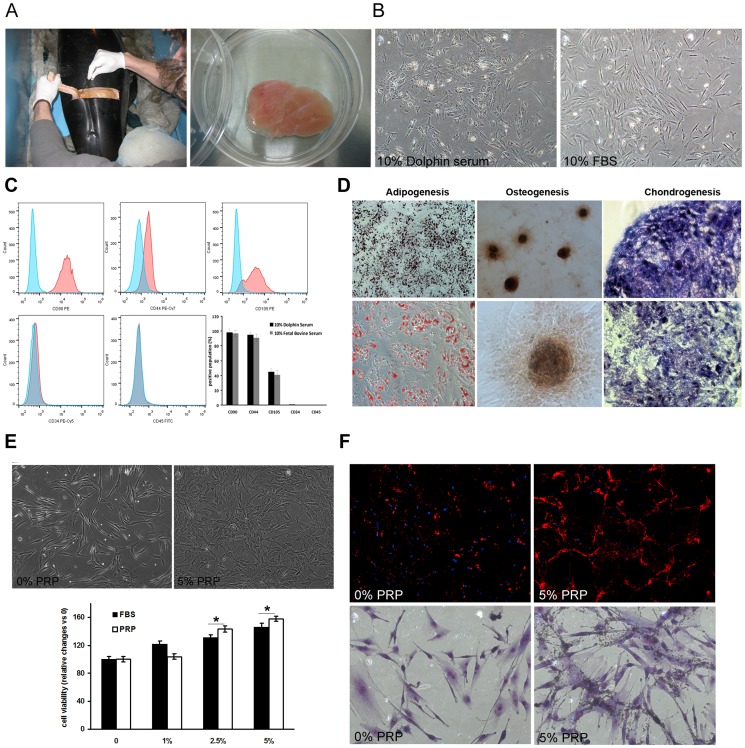
Dolphin PRP induces proliferation and phagocytic activity of dolphin ASCs. (A) Adipose tissue was collected from the postnuchal fat pad from recent postmortem wild striped dolphins (*Stenella coeruleoalba*) (n = 2) and dolphin ASCs were derived and characterized. (B) Dolphin ASCs are plastic adherent and are able to be cultured in the presence of both 10% FBS and 10% dolphin serum. The morphology of ASCs treated with 10% dolphin serum appeared less elongated and senescent compared to those cultured with 10% FBS. (C) Dolphin ASCs were positive for mesenchymal cell markers CD90, CD44, and CD105 and were negative for hematopoietic cell markers CD34 and CD45. The histograms for CD90, CD44, and CD105 show the shift in the positive population in pink versus the non-stained sample in blue. CD34 and CD45 did not show positive reactivity, thereby confirming that the putative ASCs are indeed of mesenchymal origin. (D) Dolphin ASCs were capable of tri-lineage mesenchymal differentiation. ASCs were differentiated under standard *in vitro* conditions to adipocytes (Oil Red O staining), osteocytes (Alizarin Red staining) and chondrocytes (Alcian blue staining). (E) Dolphin ASCs treated *in vitro* with 2.5 or 5% dolphin PRP exhibited significantly increased proliferation, while those treated with 1% PRP were not different than controls. Proliferation rates in ASCs treated with the same concentrations of FBS were similar but significantly lower at 2.5 and 5% compared to PRP. Morphologically there was an increase in the number and density of ASCs cultured with 2.5 or 5% PRP compared to controls. Representative images of dolphin ASCs treated with 0 or 5% dolphin PRP are shown. Results of MTS assays are mean ± SD of colorimetric signal from treated cells in the presence of 1, 2.5, or 5% PRP compared to the absence of PRP. Every condition was assayed in quadruplicate in three different experiments for both lines of dolphin ASCs. (F) In addition to inducing proliferation of ASCs, treatment with 5% PRP stimulates phagocytic activity in dolphin ASCs. Red fluorescent microspheres were highly phagocytosed by ASCs in the presence of PRP compared to those without PRP (upper panels). Similarly, when fixed and stained with Giemsa, there were clearly more ASCs indicating increased proliferation. Also visible are the increased number of microspheres which have been phagocytosed within the ASCs treated with 5% PRP compared to fewer microspheres inside untreated ASCs. Asterisks denote significant difference compared to controls (0% or PRP or FBS) * *P*<0.05.

The adipose tissue from the postnuchal fat pad dissected from recently postmortem (0±0.5 days) cadaveric dolphins (Figure 3A) was subjected to ASC isolation and characterization.

The putative dolphin ASCs were characterized according to the criteria put forth by the International Society for Cellular Therapy [30], and met all the criteria for status as mesenchymal stem cells. The ASCs were adherent to plastic culture dishes either growth in Dolphin autologus serum or in Fetal Bovine Serum containing medium (Figure 3B). FACS analysis of mesenchymal cell surface antigen-specific markers CD90 and CD44 was>98%, almost half of this population also showed CD105 reactivity, and a lack of expression of the hematopoietic antigens CD34 and CD45 (Figure 3C). Finally, the ASCs were induced to differentiate to adipocytes, osteocytes and chondrocytes under standard *in vitro* differentiation protocols (Figure 3D).

### Dolphin PRP stimulates proliferation and activates phagocytosis in dolphin ASCs

Dolphin ASCs treated with 2.5 or 5% dolphin PRP exhibited significant increases in cell proliferation as assessed by cell viability assays (MTS assay), while treatment with 1% PRP did not lead to significant changes in proliferation compared to controls (Figure 3E). FBS, used as a positive control, showed a similar pattern of proliferation as that seen with PRP (Figure 3E). Morphologically, the photomicrographs clearly illustrate an increase in the number and density of ASCs in the presence of 5% PRP compared to non-treated ASCs (Figure 3E).

PRP also activates additional ASC properties, such as phagocytic activity. Dolphin ASCs cultured in the presence of 5% PRP exhibited enhanced ability to phagocytose red fluorescent 2 µm microspheres which were added to the culture system (Figure 3F, upper panels). The enhanced phagocytic activity of ASCs induced by PRP is also clearly demonstrated by Giemsa staining. There is increased proliferation of ASCs treated with PRP and several microspheres have been phagocytosed and are unmistakably visible within the ASCs (Figure 3F, lower panels).

## Discussion

The regenerative capability of multiple growth factors found in platelets has been harvested and used in the form of PRP for regenerative purposes in multiple species for several years. However, to our knowledge, no studies have utilized dolphin PRP. ASCs in dolphins were only recently identified [27], thus there is an obvious paucity of information regarding their characterization. In this study, we have developed a simple and reproducible centrifugation protocol that yields high quality PRP which is able to induce proliferation of dolphin ASCs *in vitro*. For the first time we have identified dolphin platelets and characterized them by transmission electron microscopy and measured the levels of three major growth factors in dolphin PRP. Furthermore, we derived and characterized dolphin ASCs and demonstrated that dolphin PRP is able to induce proliferation and activate phagocytotic activity of ASCs *in vitro*.

Especially in aquariums and zoos throughout the world, the need for simple, effective, and standardized procedures to treat non-experimental animals is a necessity. Injuries caused by enclosures or repetitive movements to animals in captivity require immediate attention to avoid prolonged and chronic damage to the tissue. PRP has been linked with improvements in wound regeneration, such as reduced healing time, in multiple tissues and in several species [3], [15]–[18]. However, detailed procedures for collection and isolation of PRP had never before been documented in dolphins. Therefore separation conditions by single centrifugation of sodium citrate collected whole blood were initially based on those commonly employed for use in other mammals, specifically humans and dogs, which have been more extensively described [7]. A commonly used condition for PRP collection is 1870 rpm (460× g) for 8 min [3], therefore a series of preliminary studies were performed using this condition. To identify platelets by FACS analysis the platelet glycoprotein CD41 was employed. CD41 constitutes the alpha subunit of a highly expressed platelet surface integrin protein and appears on the platelet surface before activation thereby rendering it a reliable marker of platelets [31]. Evolutionary analyses demonstrate that dolphins share common gene and protein expression patterns with humans and that dolphin physiology may be a reliable model for studying human disease [32], [33], Therefore, although commercial dolphin antibodies are not currently available, a CD41 antibody of human origin was utilized and indeed it did recognize dolphin platelets (Figure 1B). A similar profile for antibody binding and cellular size was found for the CD41+ population in both dolphins and humans. Of note, in the FACS analysis of both human and dolphin whole blood, there was a population of CD41+ cells found within the leukocyte-expected size spectrum that most likely aggregated with larger cells such as leukocytes during the antibody incubation process. The results of platelet concentration at 1870 rpm (460× g) for 8 min were unexpectedly low as confirmed by FACS acquisition of CD41+ cells (preliminary data not shown, however this condition is shown in Figure 1C). Therefore, a range of centrifugal conditions were examined in order to optimize conditions for PRP isolation, ranging from 900–4000 rpm (106–2102× g) for 3–8 min. The initial evaluation was performed by qualitative analysis via optical microscopy of blood smears (data not shown) and subsequently quantified by FACS analysis. The most effective centrifugation condition, which yielded the highest absolute number of platelets and the highest platelet concentration compared to whole blood, was the lowest speed and duration, i.e., 900 rpm (106× g) for 3 min (Figure 1C). Increasing centrifugal force had an inverse relationship with absolute platelet number. At 900 rpm (106× g) for 3 min, the platelet concentration doubled compared to whole blood, however using the same force but doubling the duration of centrifugation (6 min) lead to a slight decline in platelet concentration compared to whole blood (Figure 1C). An alternative explanation is that in dolphins, platelet integrity could be compromised with increasing centrifugal forces and increasing time of centrifugation. Likewise, in humans, the concentration of sP-selectin, which is a marker of platelet activation and growth factor release [34], increases with elevated centrifugal forces (800–1600× g) [35], indicating compromised integrity of the platelets. Thus, platelet integrity is critical for proper growth factor release and responsiveness and for PRP to be an effective treatment in any species, platelets need to be intact, non-activated, and be able to secrete growth factors upon controlled activation. Therefore, high centrifugal forces applied to dolphin whole blood may compromise the integrity of platelets and render them inadequate for high quality PRP isolation due to loss of platelet integrity. This error in the technical step of PRP preparation and isolation may help to explain some reports indicating non-beneficial effects of PRP treatment [36]. Furthermore, our initial studies utilizing fewer revolutions (<900 rpm) were unable to concentrate the platelets to levels different than that of whole blood, i.e., lower centrifugal forces did not produce PRP in dolphin blood samples. Likewise, centrifugation at 900 rpm for a shorter duration (1 or 2 min) was unsuccessful in yielding sufficient plasma to collect and process. Therefore, to effectively isolate platelets for use in PRP-associated treatments with dolphin blood it is important to maintain the proper balance between speed and the duration of centrifugation.

To our knowledge, there are no data describing the morphology of dolphin platelets. Therefore, to characterize their morphology and further investigate the activity of platelets *in vitro* we have for the first time, described the ultra-structure of dolphin platelets via transmission electron microscopy and compared them to human platelets (Figure 2). Dolphin platelets have a larger area than human platelets (Figure 2A,B), likewise measurements across the x- and y-axes of platelets demonstrate that the distance of both of these measurements is longer in dolphin platelets than human platelets (Figure 2A,C). The larger sized platelets found in dolphin whole blood may explain the need for a reduction in centrifugation force and duration to obtain PRP compared to the optimal centrifugation conditions for human whole blood. Although dolphin platelets are larger in size, they contain similar numbers of alpha granules as human platelets (Figure 2D), although the alpha granules in dolphin platelets are also larger than those of human platelets. These observations may indicate that platelets from dolphins are extremely similar to those of humans but that components are proportionally larger. Further platelet analysis and research is needed to make decisive conclusions.

Multiple growth factors secreted by platelets are important for numerous functions including tissue regeneration, reducing inflammation, and wound healing. To our knowledge, no previous study has quantified growth factors in dolphin platelets, therefore it was necessary to evaluate essential growth factors that are associated with the improved regenerative ability of PRP and to determine if these factors are present and active in putative dolphin PRP. Therefore, three growth factors in particular were selected and measured by ELISA; platelet-derived growth factor (PDGF-BB), transforming growth factor beta (TGFβ), and vascular endothelial growth factor (VEGF-A). PDGF is known to induce proliferation of undifferentiated mesenchymal cells and some progenitor populations [37]. The tissue repair mechanisms induced by PDGF-BB appear to involve fibroblast proliferation, collagen production, and neovessel formation [38]. Several phase III human clinical trials have demonstrated the efficacy of PDGF [39], and topical application is safe, well-tolerated, and improves healing of chronic diabetic foot ulcers [40]–[42]. Dolphin PRP contained similar concentrations of PDGF-BB as in humans, hence PRP treatment in dolphins may also provide similar regenerative effects for soft tissue injuries associated with acute or chronic wounds which often occur in this species in captivity. In addition, wound repair requires the reestablishment of a functional vascular network. One of the most potent pro-angiogenic agents is VEGF, which binds the VEGF receptor on vascular endothelial cells [43], and initiates the MAPK signaling pathway which induces angiogenesis [44], [45]. TGFβ is vital for cutaneous regeneration after injury [46], and induces fibroblast proliferation and migration into the site of injury [47]. TGFβ also triggers the production of a collagen-rich matrix, which induces differentiation of fibroblasts into myofibroblasts which promote wound closure by acquiring contractibility and expressing α smooth muscle actin [47], [48]. Thus, TGFβ is a critical component in the regenerative action of PRP. Interestingly in dolphins, concentrations of VEGF-A and TGFβ in platelets were significantly reduced compared to humans.

A recent study successfully established the use of ultrasound-guided liposuction to obtain ASCs from the postnuchal fad pad of bottlenose dolphins in captivity [27]. To circumvent surgical procedures and potential injuries to captive dolphins, adipose tissue was obtained from the postnuchal fat pad of two recent (0±0.5day) postmortem striped dolphins found along the eastern Spanish Mediterranean coast and two separate ASC lines were produced. The International Society for Cellular Therapy [30] recommends that a minimum of three criteria must be met to effectively characterize multipotent mesenchymal stromal cells, also known as mesenchymal stem cells. Even though these criteria were designed as a guide for the characterization of human tissue, we have followed these basic guidelines to characterize dolphin ASCs and also added further levels of characterization confirming the successful derivation of dolphin ASCs. The first criterion to define ASCs is that they are adherent to plastic. Our culture system has clearly shown this to be true about the dolphin ASCs that were isolated as shown in Figure 3B. In addition to culturing the putative dolphin ASCs in 10% FBS, we found that 10% dolphin serum was also effective in maintaining dolphin ASC cultures. The second major characterization criterion is that these cells express specific surface antigens as measured by flow cytometry. The putative dolphin ASCs exhibited greater than 95% positivity for both CD90 and CD44 (Figure 3C). It is equally important to exclude the possibility of heterogeneous cell populations within the putative ASC population by identifying the lack of expression, or negative markers, of mesenchymal stem cells. CD45, a pan-leukocyte marker, and CD34, a primitive hematopoietic progenitor and endothelial cell marker, are the cells most likely to be found in mesenchymal stem cell cultures [30], [49]. As illustrated in Figure 3C, the dolphin ASCs lacked CD34 and CD45 expression. The third criterion and the most unique property of mesenchymal stem cells is that they must have the capacity for tri-lineage mesenchymal differentiation, that is, they must be able to differentiate *in vitro* to osteocytes, adipocytes, and chondrocytes. As shown in Figure 3D, we have demonstrated this under standard *in vitro* differentiation conditions by staining with Alizarin red (osteocytes), Oil Red O (adipocytes), and Alcian Blue (chondrocytes). Accordingly, taken together the data unequivocally demonstrate that the mesenchymal stromal cells obtained from the postnuchal fat pad of dolphins are indeed adipose-derived mesenchymal stem cells.

Cells are usually cultured *in vitro* using a serum-based component such as FBS. In addition to culturing the dolphin ASCs in 10% FBS, we found that 10% dolphin serum was also effective in maintaining the cells in a multipotent state. An interestingly observation was that cells treated with 10% dolphin serum were morphologically distinct to those treated with 10% FBS, appearing almost senescent.(Figure 3B). A possible explanation to why these cells appear different when cultured in serum from different sources is that in dolphins the coagulation cascade is markedly prolonged compared to humans. Dolphins, as well as killer whales, lack factor XII which is important in blood clotting [50], [51]. Multiple additional physiological adjustments are part of the dive response in marine mammals, and while such changes favor blood coagulation in terrestrial mammals, these adaptations in dolphins allow them to thrive and hunt at depth and high pressure. Likewise, in the laboratory it was difficult to effectively separate the serum fraction from the erythrocyte fraction after centrifugation of dolphin whole blood, whereas human blood samples collected in the same coagulation tubes yielded two distinct and easily separable fractions, the upper serum and the lower erythrocytes. Therefore, there is the possibility that the 10% dolphin serum which was utilized for cell culture actually contained a lower percentage of serum and consequently reduced concentrations of the normal complement of growth factors and hormones found in fetal bovine serum. This may help explain the change in the morphology of the dolphin ASCs cultured with 10% dolphin serum compared to the normal appearance of those cultured with 10% FBS.

An array of *in vitro* functional experiments utilizing the dolphin ASCs were performed to determine if dolphin PRP was able to stimulate proliferation of dolphin ASCs. Similar results from both cell lines confirm proper ASC function in culture and impart the potential of a combinatorial treatment for improved wound healing applications. Quantification of cell viability assays (MTS assay) demonstrated that there was a dose-response increase in cell proliferation of dolphin ASCs when treated with dolphin PRP (Figure 3E). A significant increase in cell viability was observed when dolphin ASCs were treated with 2.5 or 5% dolphin PRP. Consistent with the quantified significant increase in cell proliferation from the MTS assay, visual morphological inspection of the cells treated with 5% PRP revealed an increase in cell number compared to untreated controls (Figure 3E, upper panels). Thus, in the presence of dolphin PRP, dolphin ASCs proliferate at an increased rate. These data support the fact that even a small percentage of high quality dolphin PRP is able to stimulate proliferation of dolphin ASCs. A similar response in cell viability was seen between ASCs treated with PRP or FBS. These data are encouraging for non-xenogenic and autologous tissue and/or cell transplant applications and regenerative medicine interventions in dolphins.

Furthermore, the viability of ASCs treated with 10% FBS was not different than cells treated with 5% PRP, although the morphology was slightly different (Figure 3B, E). ASCs when cultured with dolphin PRP show a morphology that appears to be pre-adipocytic. It may be thought that the change in ASC morphology as seen in ASCs treated with 5% dolphin PRP might indicate that the ASCs could be differentiating down a specific lineage pathway and potentially losing multi-potent regenerative capacity. However, this was not the case because PRP did not condition the differentiation potential in any of the three directed differentiation lineages (adipocytes, osteocytes, or chondrocytes). No differences were detected in any of the differentiation processes regardless of the presence or absence of PRP (data not shown).

Thus, for the first time, we have shown that dolphin PRP induces proliferation of dolphin ASCs. This demonstrates that dolphin PRP contains the same or similar active growth factors with analogous proliferative ability as PRP from other mammals including humans. The regenerative capacity of the growth factors found in PRP is an excellent source for assisting in shortening the recovery time of open wounds and various tissue injuries in several mammals. Dolphins in particular are quite remarkable in their ability to recover from deep tissue wounds. Dolphin blubber contains organohalogens which exhibit antimicrobial properties and antibiotic activity [52], [53], likewise, isovaleric acid, another antimicrobial compound found in dolphin blubber, may help control microbial growth within and around damaged tissues [1]. Moreover, PRP has antimicrobial properties both *in vitro* and *in vivo*; seven antimicrobial peptides have been isolated from human platelets [54], [55]. Therefore, both ASCs and PRP from dolphins may contain innate antibacterial properties which favor and accelerate the recovery of damaged tissue.

In addition, PRP exerts anti-inflammatory properties thereby aiding in the reduction of pain associated with tissue injuries [56]. Mesenchymal stem cells have the capacity to modulate the immune system via a plethora of mechanisms (reviewed in [57]), and phagocytosis is the first step in triggering host defense and inflammation. Dolphin PRP appears to activate the phagocytic activity in dolphin ASCs as evidenced by their enhanced ability to phagocytose red fluorescent microspheres (Figure 3F, upper panels). The enhanced phagocytic activity of ASCs induced by PRP was also revealed by Giemsa staining, where there was increased proliferation and higher density of ASCs and increased phagocytosis of microspheres (Figure 3F, lower panels). Taken together, *in vitro* manipulation of dolphin ASCs with dolphin PRP may provide an exciting combination therapy for regenerative medicine in this species. Further studies are essential for improvements in basic understanding of dolphin ASCs and PRP and should aid in future veterinary interventions in aquatic medicine.

In summary, the findings presented in this study demonstrate that PRP collection and isolation containing high quality, intact, non-activated platelets from dolphin whole blood requires low centrifugal force and duration. Morphological measurements show that dolphin platelets are larger than human platelets and contain similar numbers of growth factor-containing alpha granules. In addition, dolphin ASCs were derived and characterized from adipose tissue obtained from the postnuchal fat pad of recently deceased wild striped dolphins. These ASCs are plastic adherent, show positive cell-surface antigen expression of CD90 and CD44 and lack expression of CD45 and CD34, and are also capable of tri-lineage mesenchymal differentiation to osteocytes, adipocytes, and chondroctyes *in vitro*. Moreover, dolphin PRP is able to induce proliferation of dolphin ASCs *in vitro*, demonstrating that dolphin PRP contains active growth factors. Potential treatments using dolphin PRP alone may have the capacity to treat injuries such as soft tissue wounds, however a combination therapy of dolphin ASCs and dolphin PRP either applied at the same time or ASCs treated with PRP *in vitro* and then transplanted to the injury site, may have incredible potential to treat injuries of mesenchymal origin, such as soft tissue, bone, cartilage, or tendon in dolphins. Furthermore, these findings most likely will be able to be extrapolated and applicable to other Cetaceans and marine mammals.
